# Echocardiographic Evaluation of Ventricular Function—For the Neonatologist and Pediatric Intensivist

**DOI:** 10.3389/fped.2018.00079

**Published:** 2018-04-04

**Authors:** Cécile Tissot, Yogen Singh, Nicole Sekarski

**Affiliations:** ^1^Centre de Pediatrie, Clinique des Grangettes, Geneva, Switzerland; ^2^Addenbrooke’s Hospital, Cambridge University Hospitals NHS Foundation Trust, Cambridge, United Kingdom; ^3^Pediatric Cardiology Unit, Department of Pediatrics, Centre Hospitalier Universitaire Vaudois (CHUV), Lausanne, Switzerland

**Keywords:** echocardiography, point-of-care, intensive care, pediatric, neonatology, ventricular function, functional echocardiography, bedside cardiac ultrasound

## Abstract

In the neonatal and pediatric intensive care setting, bedside cardiac ultrasound is often used to assess ventricular dimensions and function. Depending upon the underlying disease process, it is necessary to be able to evaluate the systolic and diastolic function of left and or right ventricles. The systolic function of left ventricle is mostly assessed qualitatively on visual inspection “eye-balling” and quantitatively by measuring circumferential fraction shortening or calculating the ejection fraction by Simpson’s planimetry. The assessment of left ventricular diastolic function relies essentially on the mitral valve and pulmonary venous Doppler tracings or tissue Doppler evaluation. The right ventricular particular shape and anatomical position does not permit to use the same parameters for measuring systolic function as is used for the LV. Tricuspid annular plane systolic excursion (TAPSE) and S′ velocity on tissue Doppler imaging are more often used for quantitative assessment of right ventricle systolic function. Several parameters proposed to assess right ventricle systolic function such as fractional area change, 3D echocardiography, speckle tracking, and strain rate are being researched and normal values for children are being established. Diastolic function of right ventricle is evaluated by tricuspid valve and hepatic venous Doppler tracings or on tissue Doppler evaluation. The normal values for children are pretty similar to adults while normal values for the neonates, especially preterm infants, may differ significantly from adult population. The normal values for most of the parameters used to assess cardiac function in term neonates and children have now been established.

## Introduction

Cardiovascular instability is very common in sick neonates and children needing intensive care treatment (1, 2). Many neonatal and pediatric pathologies or interventions for their management in the intensive care may affect cardiac function. Clinical examination, routine intensive care monitoring, and biochemical parameters lack sensitivity and specificity to assess cardiac function, which can be easily assessed on echocardiography (3, 4). In conjunction with clinical examination and routine cardiovascular monitoring, bedside echocardiography is an invaluable tool allowing for real-time, rapid, and reliable diagnostic information necessary for the clinical decision making in sick neonates and children (3). Evaluation of systolic and diastolic ventricular function allows targeted therapeutic interventions such as introduction or escalation of inotropic support in presence of systolic dysfunction or increase in filling pressure with lusitropic therapy when there is diastolic dysfunction (3, 5).

In the last decade, there has been a great interest among the neonatologists and pediatric intensivists in using bedside cardiac ultrasound. In fact, now it is being used in most of the neonatal and pediatric intensive care units in the Western world. There is no structured training program specifically designed to train the neonatologists or intensivists in bedside point-of-care echocardiography (6, 7).

With advancement in technology, echocardiographic evaluation of cardiac function is changing rapidly and new promising techniques are being researched. This review article provides an overview of the basics for ventricular function assessment on echocardiography which can be applied by the neonatologists and pediatric intensivists in their clinical practice. A detailed description of the advanced techniques to assess cardiac function which may be difficult to use on bedside echocardiography in the intensive care setting is beyond the scope of this article. The reference range and normal values have been provided, where possible, as certain values on echocardiography depend on the age, weight and height of the child. We recommend using *Z* scores (http://parameterz.blogspot.nl/or cardio-Z app) for precise accuracy for the individual patient.

## Measurement of Cardiac Dimensions

Measurement of cardiac dimensions is essential in the evaluation of ventricular function in newborns and children. The size of cardiac chambers and great vessels increase progressively with growth—reaching half of the adult size at birth, 75% of the adult size by 5 years, and 90% of the adult size by 12 years of age (8).

**Figure 1:**
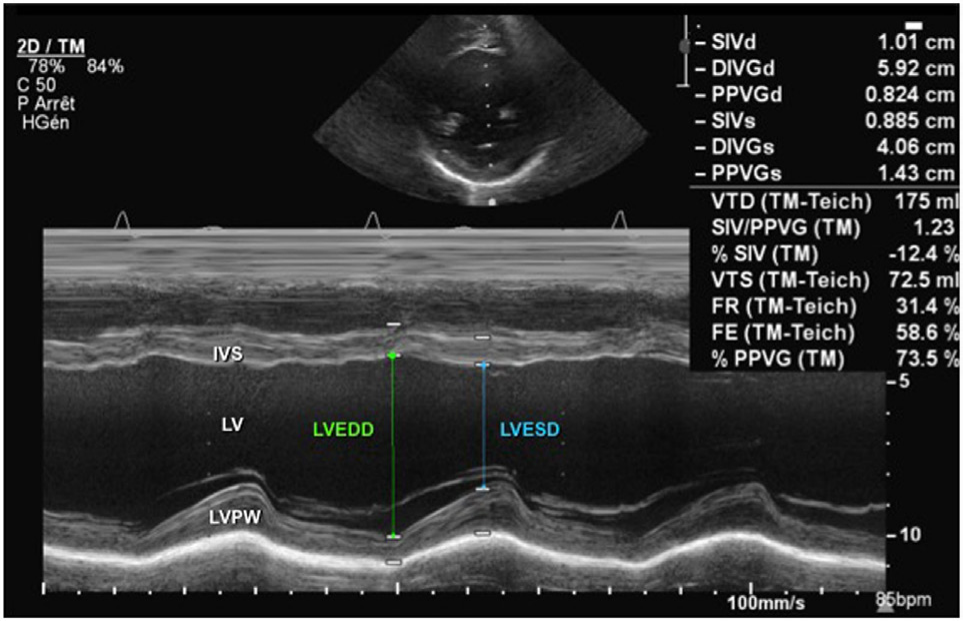
M-mode echocardiography from a parasternal short-axis view showing the left ventricular cavity over the cardiac cycle (see ECG tracing) during systole and diastole. Abbreviations: IVS, interventricular septum; LV, left ventricle; LVEDD, left ventricle end-diastolic dimension; LVESD, left ventricle end-systolic dimension; LVPW, left ventricle posterior wall.

Ventricular size can be measured by M-mode and as a standard it is measured using the leading-edge to leading-edge technique. The left ventricular M-mode tracing is obtained from the parasternal long-axis (PLAX) or parasternal short-axis (PSAX) view (Figure 1). The cursor in M-mode should be placed perpendicular to the interventricular septum and posterior wall at the level of the posterior mitral valve leaflet. Uniformity of M-mode assessment site is essential because ventricular contraction is not uniform from the base to the apex. End-diastole measurement should be done at R-wave, the beginning of the QRS complex (9), and end-systole measurement should be done at end of T-wave, but taking the measurements at the largest and smallest ventricular diameter may be more pertinent.

The American Society of Echocardiography has published guidelines on performing a pediatric echocardiography and chamber quantification in children (10, 11). For the accurate measurements of cardiac chambers, leading-edge to leading-edge technique should be used. The values should be indexed to the body surface area (BSA) and then expressed as *Z* scores (12).

## Evaluation of Left Ventricular Systolic Function

Determination of systolic function is fundamental in the management of the hemodynamically unstable newborn or child. Assessment of left ventricular systolic function should include measurement of fractional shortening (FS), ejection fraction (EF) and assessment of cardiac output (CO). Various methods can be utilized to get these data.

### Qualitative Assessment

Qualitative assessment on visual inspection “eyeballing” may be done by an experienced non-echocardiographer to evaluate left ventricle function (13). Multiple echocardiography views are usually used for visual inspection and left ventricle function may be subjectively classified into: normal function (EF ≥ 55%), mild dysfunction (EF 41–55%), moderate dysfunction (EF 31–40%), and severe dysfunction (EF ≤ 30%) (14).

### Quantitative Assessment

**Figure 2:**
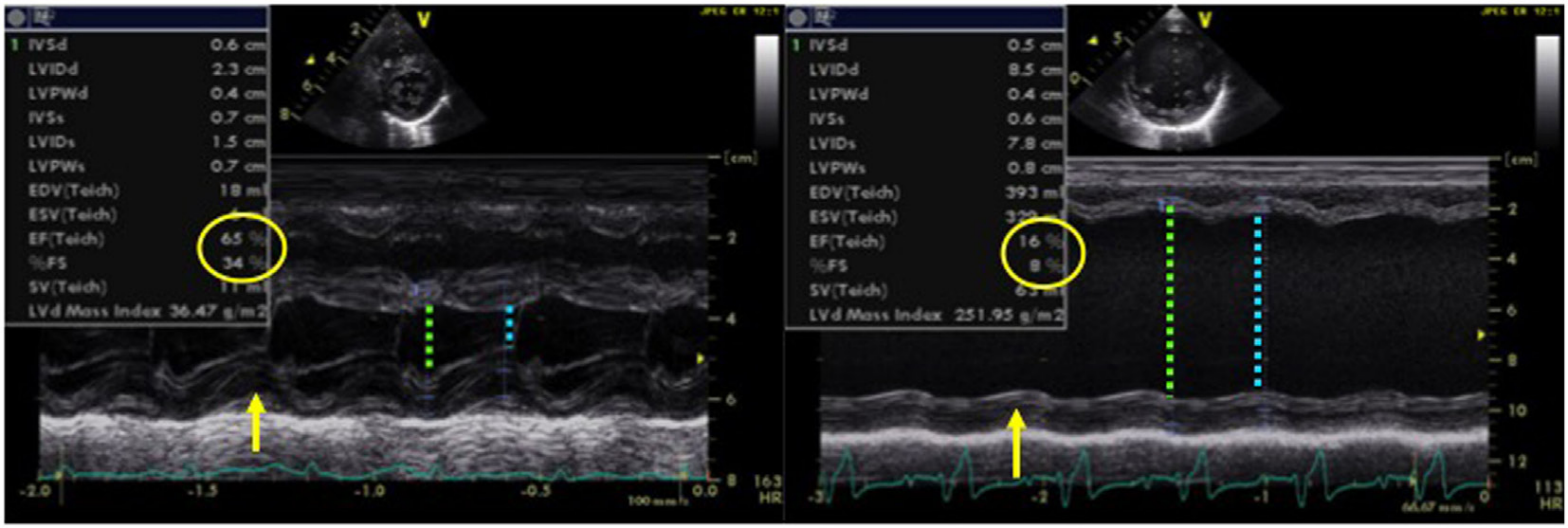
M-mode echocardiography obtained from a patient (left image) with normal left ventricular systolic function (FS = 34% and EF = 65%) and in a patient (right image) with dilated cardiomyopathy with severe left ventricular systolic dysfunction (FS = 8% and EF = 16%), LVEDD with green arrow and LVESD with blue arrow. Abbreviations: EF, ejection fraction; FS, fractional shortening; LVEDD, left ventricle end-diastolic dimension; LVESD, left ventricle end-systolic dimension.

The most commonly used parameters to assess left ventricular systolic function are FS and EF. The FS is obtained from M-mode tracings (Figure 1) or 2D imaging in the PLAX view at the tips of the mitral valve leaflets or in the PSAX view at the level of the papillary muscles. The left ventricular end-diastolic dimension (LVEDD) is measured at R-wave of cardiac cycle and left ventricular end-systolic dimension (LVESD) obtained at end of T-wave, and the FS is calculated using the following equation:

$$\text{FS }(\%)=\frac{\text{LVEDD }-\text{ LVESD}}{\text{LVEDD}}\times 100.$$

Normal values for FS in infants and children have been established and are typically between 28 and 46% (15–17). Left ventricular function may be objectively classified into the following: normal function (FS 26–45%), mild dysfunction (FS 20–25%), moderate dysfunction (EF 15–19%), and severe dysfunction (EF ≤14%) (12, 15, 17). Figure 1 demonstrates normal shortening fraction, while Figure 2 demonstrates very low reduced fraction shortening reflecting severe left ventricular systolic impairment (Figure 2). The disadvantage of this method is that it assumes a cylinder shape of the left ventricle (LV). One should be aware of the limitations. If left ventricle shape is altered, it may affect the estimation of FS leading to under- or over-estimation. The left ventricle shape may be altered in presence of congenital heart defects, change in loading conditions (preload and afterload), and affected by right ventricular (RV) dysfunction because of ventricular interdependence.

**Figure 3:**
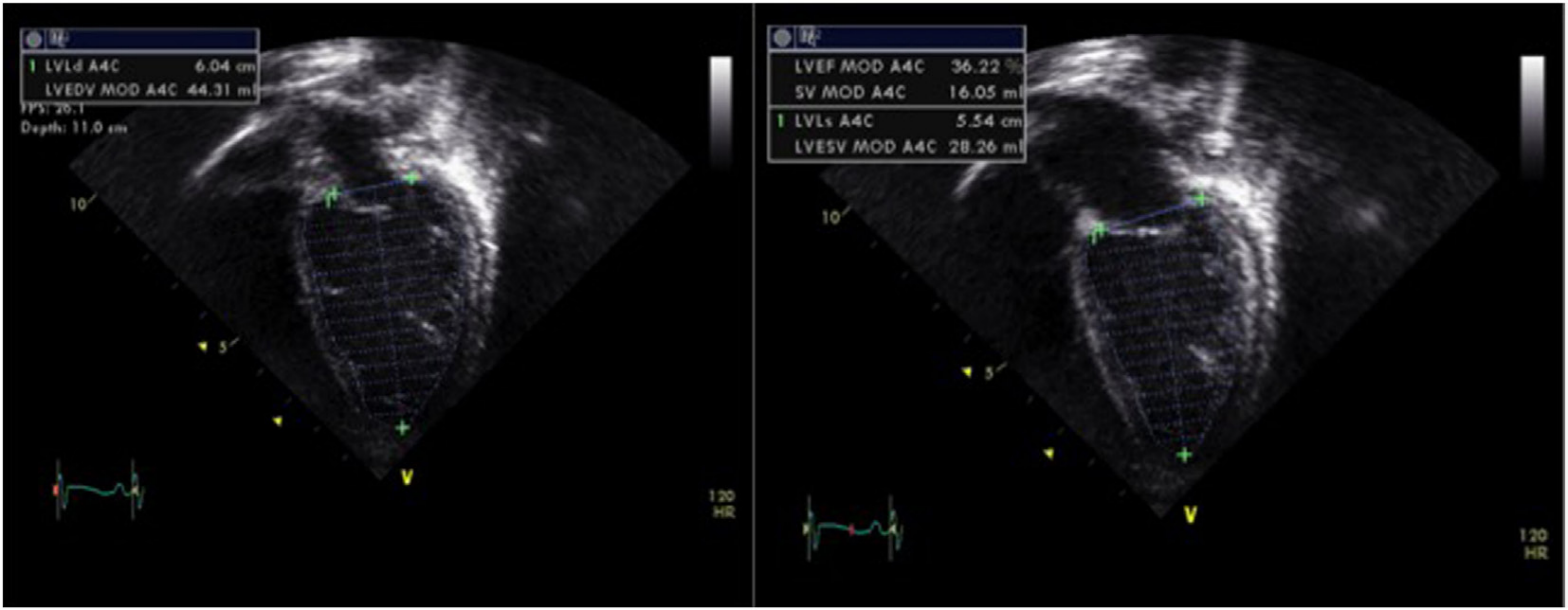
Assessment of left ventricular systolic function by planimetry using the modified Simpson’s method and allowing estimation of the ejection fraction (EF) by tracing the endocardial left ventricular border in end-diastole (LVEDV) and in end-systole (LVESV), obtained from a patient with moderate left ventricular systolic dysfunction (EF = 30%). Abbreviations: LVEF, left ventricular ejection fraction; LVEDV, left ventricle end-diastolic volume; LVESV, left ventricle end-systolic volume; SV, stroke volume.

The left ventricular function can also be assessed by calculation of the EF, which is a volumetric appraisal of ventricular fiber shortening. The best method to calculate EF is the biplane measurement of left ventricular volumes from the apical four-chamber and two-chamber views (Figure 3). The left ventricular volumes are calculated by tracing the endocardial border manually at end-diastole and at end-systole (planimetry). Modified Simpson’s method (biplane disk summation method) is the most frequently used algorithm to calculate EF in children. It uses the method of disks—left ventricle is divided into a series of parallel planes and the resultant disks are individually summed to create each volume (18, 19). The EF is calculated using the following equation:

$$\text{EF }(\%)=\frac{\text{LVEDV }-\text{ LVESV}}{\text{LVEDV}}\times 100.$$

Normal values for EF in children are between 56 and 78%. There are certain limitations to this method: especially the assumption of LV being cylindrical in shape, substantial intraobserver and interobserver variability, risk of left ventricular foreshortening, and insufficient image quality for accurate endocardial tracing (12, 18, 19). Like fraction shortening, change in left ventricular shape may alter the value of EF significantly. Intensive care patients often have sub-optimal views with difficulty in tracing the endocardial border correctly and this may be even more difficult to trace endocardial border in preterm neonates. The patients on extracorporeal membrane oxygenation or children with ventricular assist devices often need evaluation of their ventricular function, in particular during the weaning process. It is difficult to accurately assess ventricular function in an underfilled heart by calculating EF or fraction shortening; however, new advanced techniques such as tissue Doppler imaging (TDI), strain and strain rate (SR), and 3D echocardiography may be particularly helpful in these situations (discussed later).

The image quality in children on mechanical ventilator is often sub-optimal for accurate assessment of cardiac function. In these situations, alternative echocardiographic parameters may be considered to evaluate LV function.

**Figure 4:**
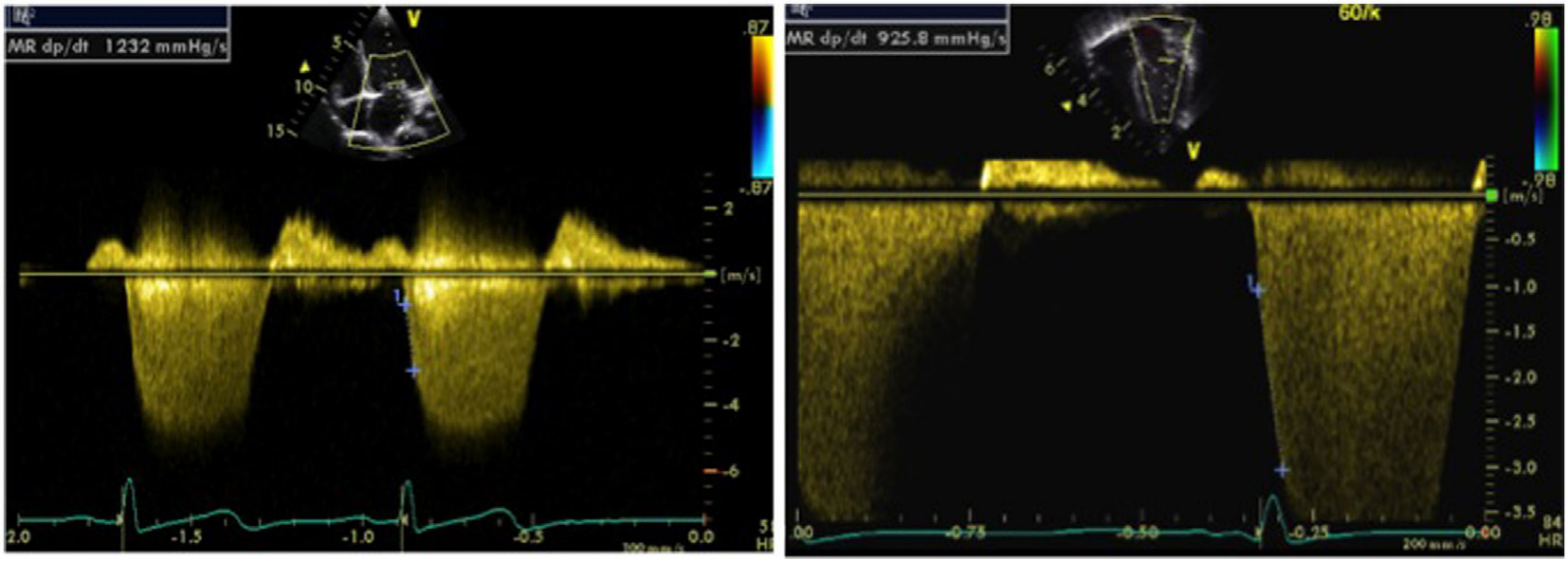
Doppler tracing from mitral regurgitation (MR) allowing measurement of the MR dP/dt max obtained between 1 and 3 m/s, on a normal patient (left image) and on a patient with left ventricular dysfunction (right image). An MR dP/dt max <500 mmHg/s is indicative of left ventricular systolic dysfunction. The same can be applied to tricuspid regurgitation (TR dP/dt max) but has to be measured between 1 and 2 m/s.

In presence of mitral regurgitation (MR), the rate of pressure rise in early systole (dP/dt max) may be used to evaluate global left ventricular contractility. MR jet velocity depends on the pressure gradient between the LV and left atrium. As there is no significant change in left atrial pressure during isovolumetric contraction (IVCT), MR dP/dt reflects left ventricular pressure changes. Using continuous wave Doppler MR spectral is acquired to calculate dP/dt: it represents time duration between change of velocity from 1 to 3 m/s on the MR spectral. The normal value of MR dP/dt is ≥1,000–1,200 mmHg/s and a value of <500 mmHg/s is indicative of severe systolic dysfunction (Figure 4). There are limitation of assessing cardiac function using dP/dt measurement—presence of MR is a pre-requisite and it is influenced by preload, afterload, heart rate, and myocardial hypertrophy (11, 12).

**Figure 5:**
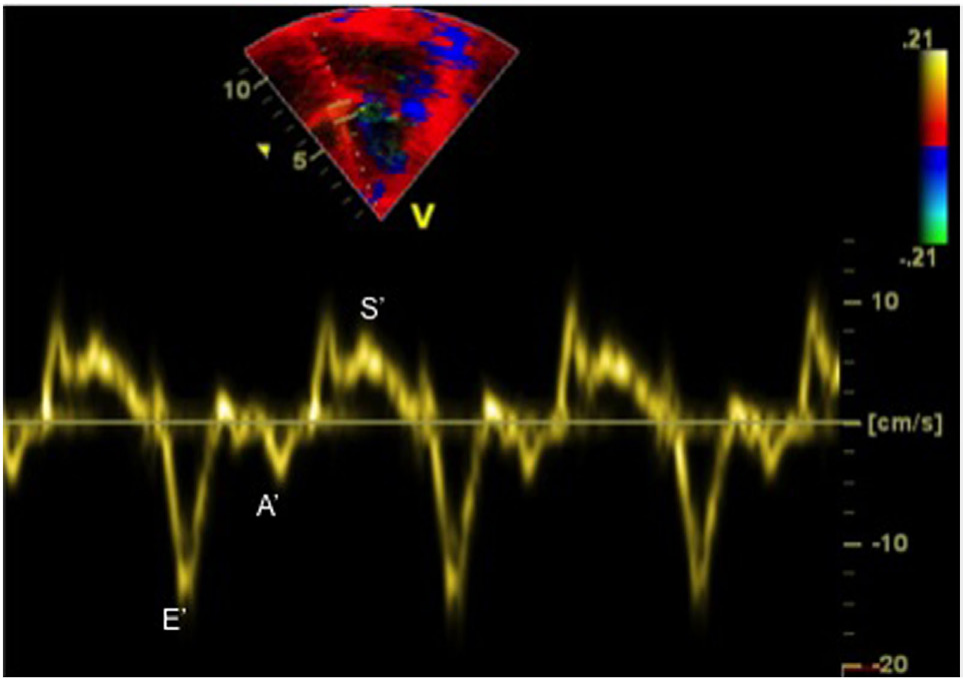
Tissue Doppler imaging tracing obtained at the septal annulus showing S′ (systolic) wave, E′ (early diastolic) wave, and A′ (late diastolic related to atrial contraction) wave.

Tissue Doppler imaging allows evaluation of myocardial velocities. Peak systolic annular velocity (S′ wave) measured at the level of the mitral annulus reflects left ventricular contractility (Figure 5). S′ value is considered as a reliable qualitative measure of global left ventricular systolic function, and the normal value in adults is ≥10 cm/s.

**Figure 6:**
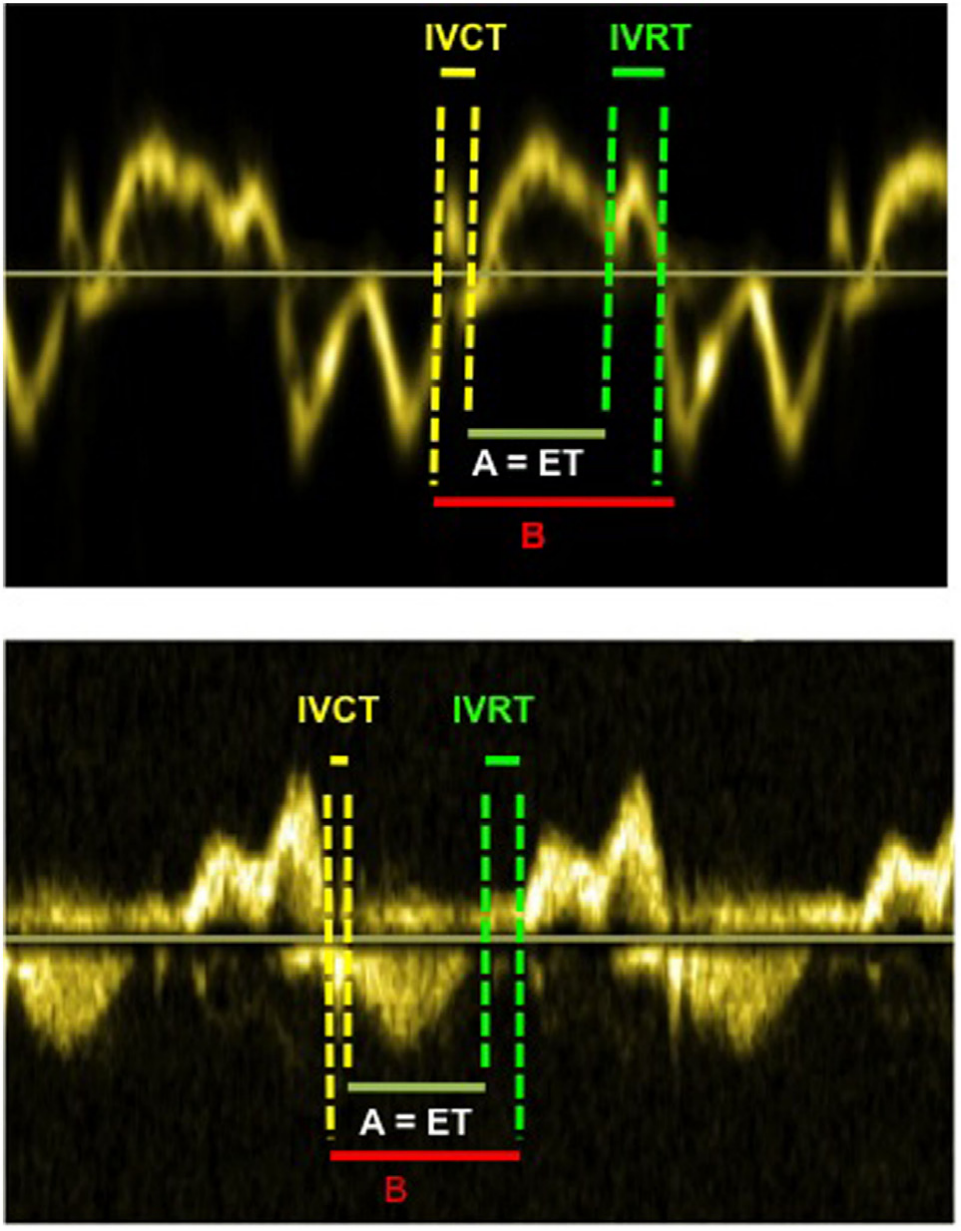
Myocardial performance index (=MPI or Tei index) obtained from tissue Doppler (above image) and from Doppler (below image). The MPI is calculated as the (IVCT + IVRT)/ET or (B − A)/A. The MPI allows evaluation of systolic and diastolic ventricular function. Abbreviations: ET, ejection time; IVCT, isovolumetric contraction time; IVRT, isovolumetric relaxation time.

The measurement of myocardial performance index or Tei index allows evaluation of both systolic and diastolic function simultaneously (20). The myocardial performance index (MPI) has been reported to be a reliable method for the evaluation of left ventricular performance in well children, although its reliability in neonates remains questionable. It is calculated from pulsed wave (PW) Doppler at the mitral and aortic valve simultaneously or using TDI tracing at the lateral mitral annulus (Figure 6), and then index is derived from the following formula:

$$\text{Tei index }(\text{or MPI})=\frac{\text{IVRT}+\text{IVCT}}{\text{ET}},$$

where IVRT is isovolumetric relaxation time, IVCT is isovolumetric contraction time, and ET is the ejection time of LV.

The Tei index value depends upon the age of child, especially during the first 3 years of life, with significantly greater values (0.4 ± 0.09) at birth and progressive reduction until the age of 3 years (14). There are no further changes from age 3 to 18 years and normal value is 0.33 ± 0.02 by pulsed wave (PW) Doppler. However, like many other parameters, it depends upon the following: angle of insonation (angle between blood flow or ultrasound Doppler waves), loading conditions, ability to get mitral and aortic Doppler in the same image, and accuracy of measurement (10, 11).

## Evaluation of Left Ventricular Diastolic Function

In the last decade, more emphasis has been on the assessment of diastolic function especially in the postoperative period in the intensive care unit. Evaluation of left ventricular diastolic function is complex and a combination of different echocardiographic markers may be used (Table 1), and a detailed description of all the techniques is beyond the scope of this review. Commonly used echocardiographic parameters useful to the intensivists in bedside echocardiography are described here.

**Table 1:** Evaluation of diastolic function using mitral inflow Doppler, pulmonary venous Doppler and mitral annulus tissue Doppler pattern with classification into normal, delayed relaxation (=grade 1 or mild diastolic dysfunction), pseudo-normal (=grade 2 or moderate diastolic dysfunction), and restriction (=grade 3 or severe diastolic dysfunction).

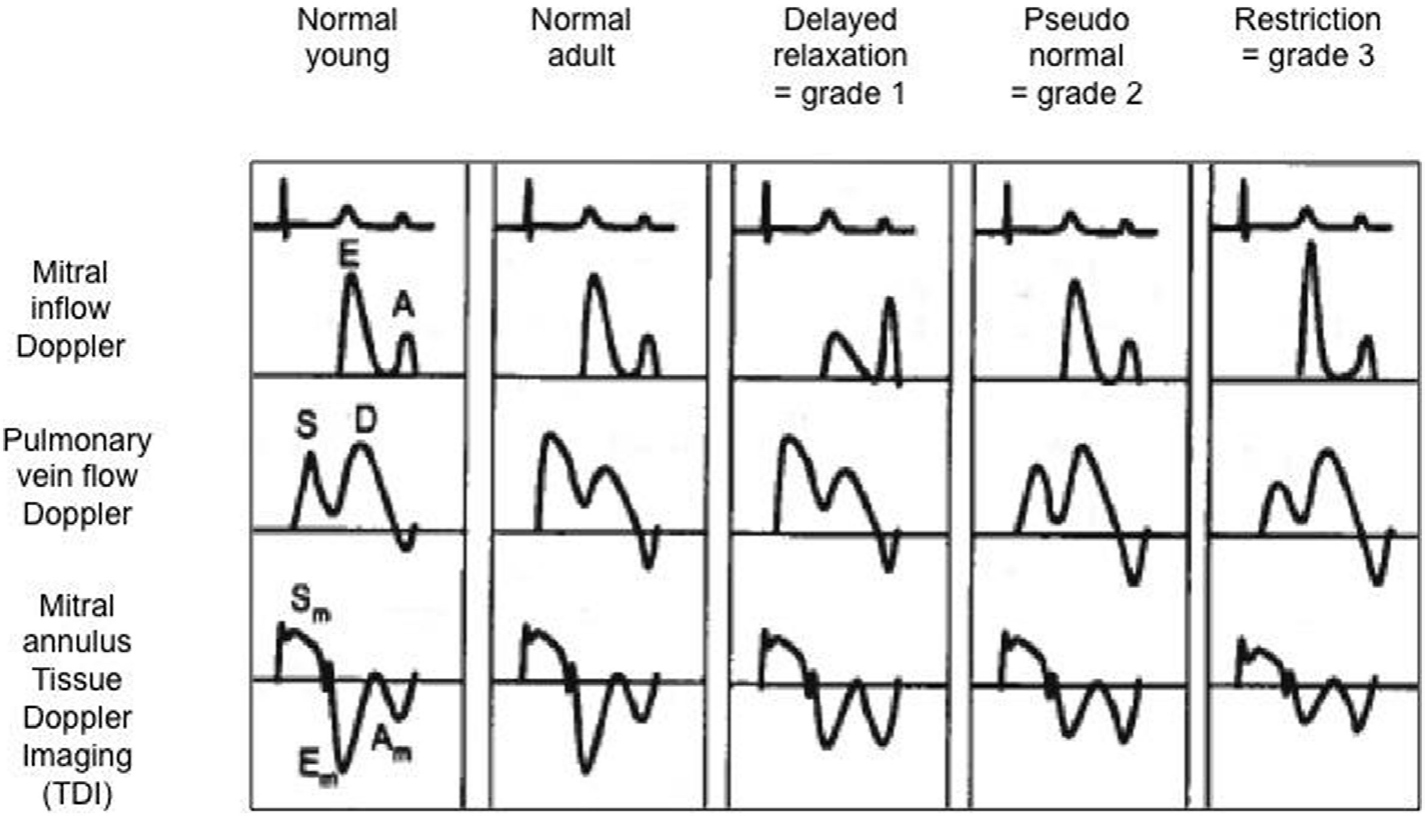

*Adapted from Tissot et al. (21)*.

*A, Doppler mitral late diastolic wave; A_m_ (ou A′), TDI late diastolic wave; D, Doppler pulmonary venous diastolic wave; E, Doppler mitral early diastolic wave; E_m_ (ou S′), TDI early diastolic wave; S, Doppler pulmonary venous systolic wave; S_m_ (ou S′), TDI systolic wave*.

**Figure 7:**
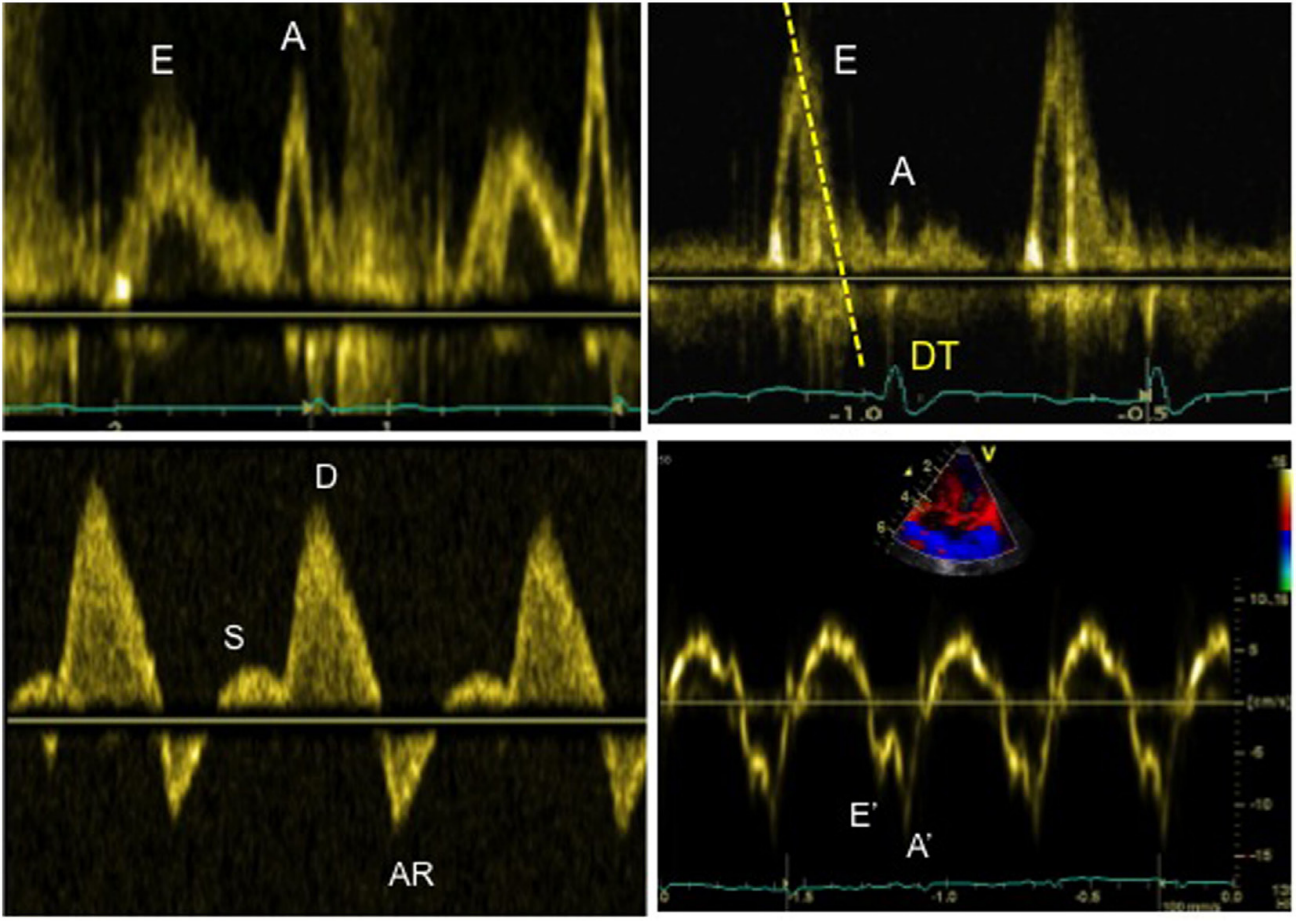
Evaluation of diastolic function in a patient with severe diastolic dysfunction: (1) mitral inflow Doppler pattern with E and A wave reversal (above left image); (2) mitral inflow Doppler pattern with sharp E wave DT (above right image); (3) pulmonary venous Doppler with prominent diastolic flow, S/D reversal and increased velocity of AR wave (below left image); (4) TDI at the mitral annulus with E′/A′ reversal and decreased E′ wave velocity <8 cm/s (below right image).

On bedside echocardiography, diastolic function can be quickly assessed by using Doppler method and studying the changes in spectral. For example, prominent pulmonary venous A wave is a marker for flow reversal into the pulmonary veins during atrial systole, and it is a hallmark of diastolic dysfunction in a non-compliant ventricle (Figure 7). The mitral inflow Doppler pattern can also be used for estimation of left ventricular diastolic dysfunction (Figure 7). This Doppler flow pattern is affected by the age, arrhythmia, conduction disturbances, and changes in loading conditions, and the clinician should take these into account while making clinical decisions based on diastolic function assessment, particularly in sick children being managed in the intensive care unit.

Quantitative assessment of left LV diastolic function may be done by analyzing Doppler spectral of mitral inflow and pulmonary veins, or TDI evaluation in the lateral mitral annulus. The pulse wave (PW) Doppler across mitral inflow is composed of two waves: an E wave representing early passive ventricular filling (preload dependent) and an A wave representing late diastolic active filling as a result of atrial contraction. The mitral *E*/*A* ratio, velocity, and deceleration time of the E wave can be altered in patients with left ventricular diastolic dysfunction (11, 12). Similarly, PW Doppler in pulmonary veins can be used to assess left ventricular function. The pulmonary venous Doppler pattern is composed of three waves: the systolic (S) wave, the diastolic (D) wave, and the AR wave related to end-diastolic flow reversal secondary to atrial contraction. The ratio of the pulmonary venous S/D waves and AR wave velocity/duration may be altered in presence of LV function (11, 12).

Mild diastolic dysfunction is characterized by an inversion of the mitral early and late diastolic E/A Doppler waves with a ratio <0.8 (12). The echocardiographic parameters of severe diastolic dysfunction of LV are as follows: mitral valve Doppler *E*/*A* ratio >2, deceleration time of the Doppler mitral E wave <160 ms, and diastolic flow predominance in the pulmonary venous Doppler pattern (S/D < 1) with prominent atrial wave reversal (AR ≥35 cm/s) (12).

The diastolic function of ventricles can also be assessed using TDI, which allows measuring peak myocardial velocities using PW Doppler. With this technique, myocardial movement velocity is measured directly rather than the Doppler velocity of blood flow across valves. Depending upon the area of interest, a pulsed Doppler cursor is placed on the myocardial wall (mitral, septal, or tricuspid annulus) and peak myocardial velocities are recorded. Three waveforms are obtained: a peak systolic wave (S′), an early diastolic wave (E′), and an end-diastolic wave (A′) produced by atrial contraction (Figure 5) (22). The S′ wave from mitral annular velocity has been shown to correlate with global left ventricular systolic myocardial function, and a value <10 cm/s is indicative of systolic dysfunction in adults (23). Evaluation of E′ and A′ waves on TDI allows estimating ventricular diastolic function. Assessing cardiac function using TDI has added benefits as it is relatively independent of preload condition, as opposed to the Doppler evaluation (24, 25).

Tissue Doppler imaging signs of diastolic dysfunction are an early mitral annular or septal E′ wave velocity <8 cm/s. The E/E′ ratio (Doppler early mitral inflow velocity/TDI early diastolic mitral annular velocity) has been shown to correlate with the pulmonary capillary wedge pressure in adults (26). The E/E′ ratio is also helpful in estimating left ventricular filling pressure (27), with a value >14 indicative of increased left heart filling pressure in adults. TDI is currently the most accurate technique to evaluate diastolic function in children, particularly in the critically ill patients where loading conditions can vary widely.

## Evaluation of RV Systolic Function

The echocardiographic assessment of the right ventricle is more difficult because of its anterior position behind the sternum and to its complex geometrical shape (28). The RV contraction differs from the LV and is determined by longitudinal shortening. There is little or no isovolumetric contraction and relaxation, and RV afterload is low (29). Guidelines for imaging the right heart in adults have been published by the ASE in 2010 (28). There are limitations of conventional echocardiographic techniques (2D, M-mode, PW Doppler) in reliably assessing RV function. Advanced echocardiographic techniques such as TDI, speckle tracking echocardiography, and 3D echo have been suggested to have added value.

Qualitative evaluation of the right ventricle (eyeball technique) has long prevailed in the regular clinical setting. Besides being echocardiographer dependent, it is imprecise and often insufficient. An approximate assessment of the RV size can be made by comparing it to the left ventricular (LV) size, and this can be categorized into the following: normal size (RV less than 2/3 of the LV size), mildly enlarged (RV more than 2/3 of the LV), moderately enlarged (RV and LV are of same size), and greatly enlarged when RV is bigger than the LV (28, 30).

Right ventricular function can also be evaluated qualitatively. The best view to RV function is PSAX sweep at the level of the papillary muscle to look at the RV free wall and the septal motion. With volume overload, the RV will become dilated and the septum flattened in diastole, while pressure overload results in septal flattening in systole (31). The different parts of the RV can be evaluated in the main echocardiographic views (32).

The dimensions of the RV can be measured in the apical four-chamber view and the RV free wall thickness in best measured in the PSAX view. Like LV, the end-diastolic and end-systolic area of RV can be measured by manually tracing the endocardial border in systole and diastole. The papillary muscle and the trabeculae should be included in the cavity. The RV volume is probably best measured on 3D echocardiography. The volume estimation using 2D technique is sub-optimal because of its complex geometry and anatomical position (33). As the RV muscle fibers are oriented in a longitudinal way, the RV function is determined by the longitudinal shortening, reason why the M-mode FS, as it is performed for the LV, will be inaccurate (28). It is therefore recommended to use other parameters to assess RV systolic function (28, 34).

**Figure 8:**
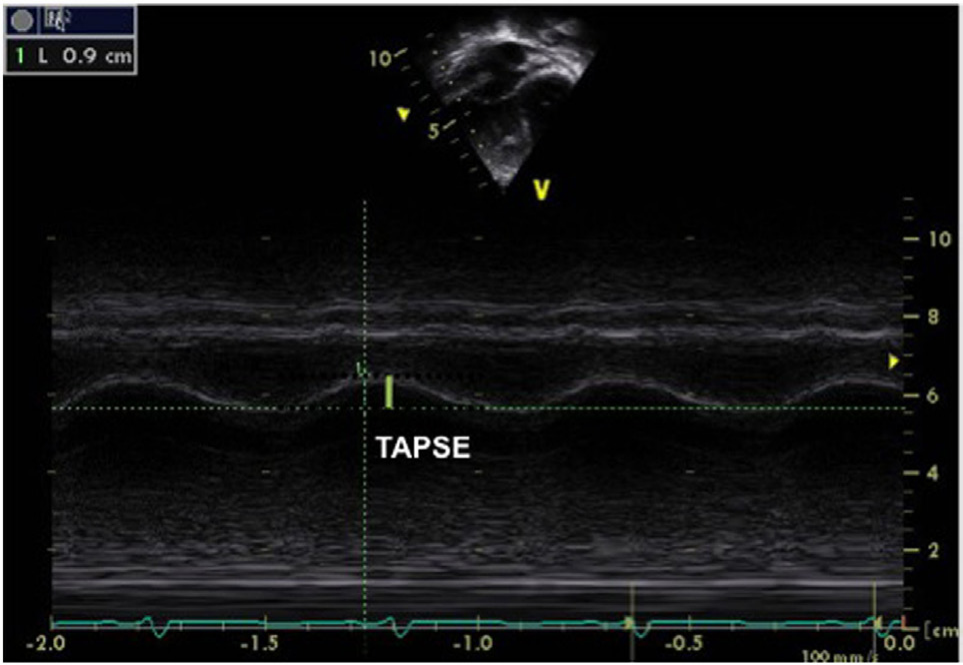
M-mode obtained from the tricuspid annulus allowing measurement of the tricuspid annular plane systolic excursion (TAPSE) in a patient with pulmonary hypertension and right ventricular (RV) dysfunction. A TAPSE value <16 cm is indicative of RV systolic dysfunction. The same can be obtained from the mitral annulus to obtain the mitral annular plane systolic excursion.

Another technique often used to assess RV function is TAPSE, which measures the excursion of the tricuspid annulus during the cardiac cycle (between early diastole and end systole). TAPSE is measured by placing an M-mode cursor through the lateral tricuspid annulus in a four-chamber view (Figure 8). This index is a reliable marker of RV longitudinal function and has been well correlated with RV EF by cardiac MRI. A value of <16 mm is adults and <10 mm in children is indicative of RV systolic dysfunction (35). TAPSE is dependent on the age and normal values have been published for healthy children (36).

As for the LV, TDI can be used to obtain a qualitative evaluation of the RV systolic function. TDI assessment is best done in the apical four-chamber view by using PW Doppler on the lateral free wall and by measuring the systolic S′ wave (35). An S′ wave velocity <10 cm/s is indicative of RV systolic dysfunction.

**Figure 9:**
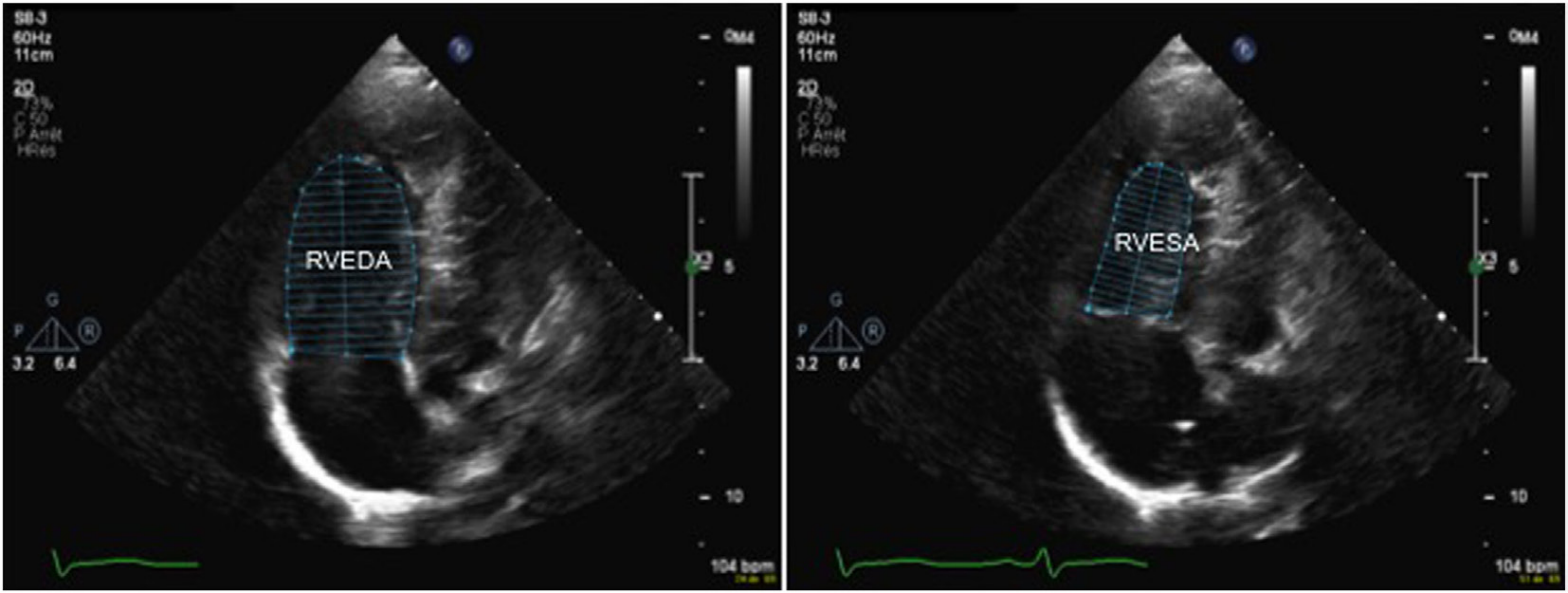
2D echocardiography from an apical four-chamber in a patient with pulmonary hypertension view allowing measurement of the right ventricular fractional area change (FAC) by tracing the endocardial border to obtain the RVEDA and RVESA. A FAC value <35% is indicative of right ventricular systolic dysfunction. Abbreviations: RVEDA, right ventricular end-diastolic area; RVESA, right ventricular end-systolic area.

Recently fractional area change has been suggested a reliable echocardiographic marker of RV function and this is calculated using the following formula:

$$FAC(\%)=\frac{\text{RVEDA}-\text{RVESA}}{\text{RVEDA}}\times 100,$$

where RVEDA is right ventricle end-diastolic area and RVESA is RV end-systolic area (Figure 9). Normal values range from 43 ± 18%, and a value <35% is indicative of RV systolic dysfunction (28).

Like for the LV, the MPI or Tei index is calculated by PW Doppler at the level of tricuspid and pulmonary valves or on TDI tracing at the lateral tricuspid annulus, with normal values of 0.24 ± 0.04 irrespective of age (36, 37).

More advanced techniques to evaluate RV function comprise 3D echocardiography and speckle tracking, a detailed description of these techniques is beyond the scope of this review.

## Evaluation of RV Diastolic Function

Right ventricular diastolic function is essentially evaluated by looking at the tricuspid inflow pattern with pulsed wave Doppler or at the lateral tricuspid annulus motion with TDI. Assessing hepatic venous flow patterns and right atrial area/volume may be helpful in assessing RV diastolic function. Similar to LV diastolic function, mild diastolic dysfunction is characterized by an inversion of the tricuspid early and late diastolic Doppler waves, *E*/*A* ratio <0.8 (4, 5). Severe diastolic dysfunction of right ventricle is characterized by a tricuspid Doppler *E*/*A* ratio >2.1, a deceleration time of the Doppler tricuspid E wave <120 ms, a Doppler hepatic venous diastolic flow predominance (S/D <1), and a ratio of tricuspid Doppler and TDI tricuspid annular annulus E/E′ >6 (30).

## Evaluation of CO and Cardiac Index

The evaluation of the CO and cardiac index on echocardiography is well established in children, and it can be easily performed on bedside echocardiography, even in sick children in the intensive care setting. CO may be decreased in presence of impaired ventricular function and assessing CO may help in targeting the specific treatment.

**Figure 10:**
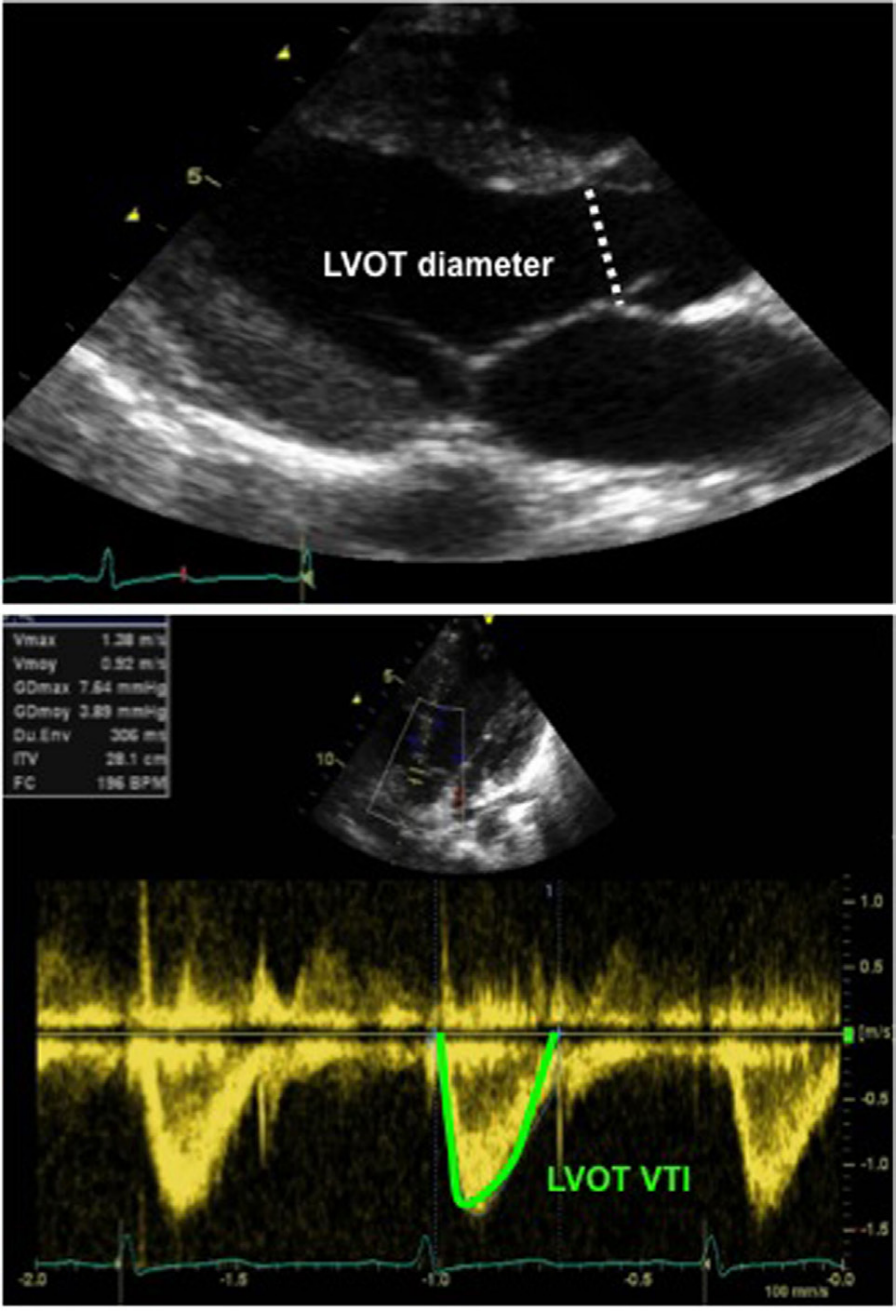
Echocardiographic assessment of cardiac output: 2D echocardiography long-axis view with measurement of LVOT diameter (above image), allowing for calculation of the LVOT CSA and Doppler tracing from an apical five-chamber view (below image) with tracing of the LVOT velocity to obtain the LVOT VTI. Abbreviations: CSA, cross-sectional area (calculated as π × *r*^2^ and *r*, radius, diameter/2): LVOT, left ventricular outflow tract; VTI, velocity time integral (or mean velocity).

The echocardiographic assessment of the CO can be obtained by measuring cross-sectional area (CSA) of the left or RV outflow tract at the level of aortic or pulmonary annulus and by measuring the velocity time integral (VTI) at the level of aortic or pulmonary valve by pulsed wave Doppler, respectively (Figure 10). The CO is calculated by using the following equation:

Cardiac Output (CO)=SV×HR=VTI×CSA×Heart Rate,

where SV is the stroke volume, VTI is the velocity time integral, CSA is the cross-sectional area, and HR is the heart rate. The cardiac index can be derived by dividing the CO by the body weight in kilogram according to the equation below (38):

$$\text{Cardiac Index }(\text{CI})=\frac{\text{VTI}\times \text{CSA}\times \text{HR}}{\text{BSA}},$$

where BSA represents the body surface area. The measurement of CO by this method is a simplistic method and involves various assumptions. Despite various limitations, the CO measured using echocardiography correlated well with other well-established methods such as cardiac MRI, cardiac catheter, and Fick’s dye dilution method. The published studies showed a bias under 10% (39).

The measurement of CO on echocardiography is pretty reliable in children without shunts, and in fact, it is fairly reliable even in neonates (39). The normal values are 150–400 ml/kg/day in infants and children. From the authors’ anecdotal experience, studying the trend on serial echocardiography may be more useful in intensive care setting to monitor the response to intervention or change in physiology. The CSA of left or right ventricle measured at aortic and pulmonary annulus is fairly constant, and heart rate can be easily obtained from the cardiac monitor. Hence, serial assessment of the VTI may help in assessing the impact of any intervention on CO in real time.

## Future Perspectives

With advances in technology, new echocardiographic techniques are being researched to assess cardiac function in neonates and children. The normal values for adults and children have been established, and initial studies have demonstrated them to be feasible in well-term neonates (11, 12). While these techniques look promising and may provide more accurate assessment of cardiac function, there are certain limitations for their use in the intensive care setting: (1) results may not be available in real time and these techniques often need post-processing making them unsuitable for making immediate clinical decisions in critically ill children; (2) special software may not be required to analyze results which may not be accessible to all the neonatologists and pediatric intensivists performing point-of-care echocardiography; (3) image acquisition and interpretation needs special expertise; and (4) image quality may be sub-optimal for accurate processing in children on mechanical ventilator, especially in neonates. These issues need addressing before these techniques can be safely used in the neonatal and pediatric intensive care clinical practice.

Two promising advanced echocardiographic techniques are on the horizons and may become available to assess cardiac function accurately in the near future.

### Myocardial Mechanics

In the recent years, myocardial mechanics have emerged as a promising tool to measure myocardial function and contractility. Strain (ε) is defined as the deformation of the myocardium relative to its original length. It is a dimensionless parameter and is expressed as a percentage. SR is defined as the local rate of myocardial deformation or strain (ε) per unit of time, and it is expressed in 1/s. Both strain and SR can be obtained from tissue speckle tracking or from TDI. The strain and SR assessment does not depend upon the ventricular geometry and loading conditions of the ventricles. The evaluation of ventricular function is limited with conventional M-mode and 2D echocardiography due to the limitation of the ventricular geometry; as it is the case with the complex morphology of the right ventricle, the altered morphology of the LV in complex congenital heart disease and the changing loading conditions that occur in the critical care settings, strain and SR may be of great help for more accurate and reliable evaluation of ventricular function (40). The normal values for left and right ventricle strain and SR are available for healthy children (41), but further research is required before it can be used in the critical care unit.

### 3D Echocardiography

Left ventricular volume, mass, and function with calculation of EF can be accurately assessed using real-time 3D (RT3D) echocardiography and is independent of geometric assumption. Acquiring the entire left ventricular volume often necessitates a wide angle mode. The analysis on 3D echocardiography allows determination of global and regional wall motion when evaluated from the base to the apex with multiple slices from different orientations. Left ventricular volume assessment by RT3D is rapid, accurate, reproducible, and superior to conventional 2D methods (42). RT3D allows manipulation of the plane to align the true long axis and minor axis of the LV, and hence avoiding oblique image planes and foreshortening of left ventricle. Measurement of left ventricular function is comparable to cardiac MRI, which is considered as the gold standard for assessing LV function (43). Given the benefits, 3D echocardiography should be increasingly used in future in the critical care setting and it should be considered as an adjunct to conventional assessment on 2D and M-mode echocardiography.

## Role of Ventricular Function Assessment in the Neonatal and Pediatric Intensive Care Unit

Assessing cardiac function is of utmost importance in critically ill neonates and children with cardiovascular instability. Bedside echocardiography may provide anatomical and physiological information in real-time helping to make clinical decisions in patients with various pathologies including: diagnosis of underlying congenital heart defect, diagnosis and management of pulmonary hypertension, shock, hypotension, hypovolemia, and perinatal asphyxia to name a few (3, 5). Functional echocardiography allows the neonatologists and pediatric intensivists in targeting specific intervention—fluid resuscitation therapy for hypovolemia, inotropes for patients with impaired cardiac dysfunction, pulmonary vasodilators in setting of pulmonary hypertension, and vasopressor therapy in conditions needing increase in systemic vascular resistance (3, 5, 44, 45).

For the specific condition, readers are advised to refer to the relevant TINEC research articles published under the same theme in *Frontiers in Pediatrics*; such as echocardiographic evaluation of patent ductus arteriosus, echocardiographic evaluation of hemodynamics, and echocardiographic evaluation of pulmonary hypertension, readers are advised to refer to the relevant TINEC research articles published under the same theme in *Frontiers in Pediatrics* (46–48).

### Special Considerations in Preterm Infants

Like older children, similar echocardiographic parameters may be used for assessing ventricular function in preterm infants. Echocardiography technique remains same, although normal values and reference range for all the parameters in preterm infants is not well established yet (11, 12). Some of the parameters may pose specific challenges. For instance, delineation of endocardium is difficult in extremely preterm infants, which makes certain techniques such as speckle tracking, measurement of EF by Simpson’s biplane planimetry difficult, and there is limited experience in 3D echocardiography in preterm infants. In fact, currently available probes for 3D echocardiography may not be suitable for extremely low birth weight infants and image quality is sub-optimal for the interpretation.

### Summary of the Clinical Practice Points for the Neonatologists and Pediatric Intensivists

A summary of the practical practice points for the neonatologists and pediatric intensivists in assessing ventricular functions is discussed below:

(a) Like any other functional assessment, use of ECG tracing during imaging acquisition is recommended for the accurate assessment of ventricular function.
(b) High-quality images should be obtained for the precise measurement of cardiac function. Any error in measurement is often multiplied in calculating algorithms. One should be aware of old saying—“garbage in, garbage out,” and this is very relevant for the imaging acquisition required for accurate functional assessment.
(c) In emergency situations, qualitative assessment on visual inspection may be used; however, there is subjectivity in interpretation.
(d) It is critical to understand the cardiac mechanics which can affect ventricular function assessment. For example, fraction shortening may not be reliably calculated in presence of paradoxical septal movements or in presence of significant right ventricle dysfunction because of ventricular interdependence.
(e) The clinician performing the scan should know the limitations of the echocardiographic parameters and their interpretation. For instance, impact of the angle of insonation on Doppler assessment and influence of loading conditions on the echocardiographic parameter being analyzed.
(f) Use multiple echocardiographic views while assessing ventricular function.
(g) The advanced echocardiographic techniques look promising, but in today’s clinical practice, there are limitations for their application in the intensive care setting.
(h) Finally but most importantly, echocardiographic assessment is only an adjunct to clinical examination and other monitoring parameters. The results from functional assessment should always be interpreted in conjunction with the clinical picture.

## Conclusion

Evaluation of cardiac function requires good image quality and multiples parameters in order to deliver accurate and reliable information. The evaluation of cardiac function can be easily performed by bedside cardiac ultrasound in the intensive care setting and physiological information gained may help in taking timely and accurate therapeutic decisions. Longitudinal assessment of cardiac function on serial bedside cardiac ultrasound may help in monitoring the patient condition and targeting the specific treatment.

While the echocardiographic assessment of left ventricular function is well established, the complex geometry of the right ventricle and ventricular interdependence make its assessment more difficult. Recently, there has been a great interest in assessing RV function using advanced techniques such as TDI, speckle tracking echocardiography, and 3D echocardiography, and the initial results from the research studies look promising.

## Author’s Note

CT is a pediatric cardiologist trained at the University Hospital of Geneva, Switzerland and in Denver, CO, USA. CT has been working as an attending pediatric cardiologist at the Children’s Hospital of Geneva (HUG) until recently and is now working in the Pediatric Center at Clinique des Grangettes, Switzerland. YS is a consultant in neonatology and pediatric cardiology at Addenbrooke’s Hospital in Cambridge, UK, and he is a course director for echocardiography in Cambridge. YS has special interest in functional echocardiography and hemodynamic assessment in children. NS is a pediatric cardiologist trained at the University Hospital of Lausanne and in Washington University, Saint-Louis, USA. NS is working as the chief of the Pediatric Cardiology unit at the Centre Hospitalier Universitaire Vaudois (CHUV). CT, NS, and YS are part of the organizing committee of the Training in Intensive Care and Neonatal Echocardiography (TINEC), a course on point-of-care echocardiography that is taking place in Lausanne, Switzerland, since January 2016.

## Author Contributions

All authors listed have made a substantial, direct and intellectual contribution to the work, and approved it for publication.

## Conflict of Interest Statement

The authors declare that the research was conducted in the absence of any commercial or financial relationships that could be construed as a potential conflict of interest. The reviewer EM and handling Editor declared their shared affiliation.
